# Dexamethasone for prevention of AMS, HACE, and HAPE and for limiting impairment of performance after rapid ascent to high altitude: a narrative review

**DOI:** 10.1186/s40779-025-00634-y

**Published:** 2025-08-11

**Authors:** Johannes Burtscher, Hannes Gatterer, Beth A. Beidleman, Martin Burtscher

**Affiliations:** 1 Institute of Sport Sciences, University of Lausanne, 1015 Lausanne, Switzerland; 2 Institute of Mountain Emergency Medicine, Eurac Research, 39100 Bolzano, Italy; 3 Institute for Sports Medicine, Alpine Medicine and Health Tourism (ISAG), UMIT TIROL-Private University for Health Sciences and Health Technology, 6060 Hall in Tirol, Austria; 4 U.S. Army Research Institute of Environmental Medicine, Natick, MA 01760 USA; 5 Department of Sport Science, University of Innsbruck, 6020 Innsbruck, Austria

**Keywords:** Altitude illnesses, Prevention, Dexamethasone, Emergency, Rescue mission, Military operation

## Abstract

Acute exposure to high altitude can cause acute altitude illnesses and is associated with impaired cognitive and physical performance. The most effective preventive strategies currently recommended include environmental acclimatization (slow ascent and/or pre-acclimatization) or pharmacological support of acclimatization using acetazolamide. However, these strategies are not practical for high-altitude exposures that require rapid and unplanned ascent, high physical and mental performance, such as rescue missions or military operations. Dexamethasone and other modulators of the glucocorticoid system take effect quickly and are effective alternatives for preventing acute altitude illnesses when rapidly ascending to high altitudes. As the efficacy of dexamethasone in preventing acute mountain illnesses remains controversial, a review of existing studies on the use of dexamethasone for the prevention of acute mountain sickness was conducted, aiming to determine the best strategy. Possible mechanisms of protection against acute altitude illnesses are discussed based on the results of clinical trials. The data indicate that dexamethasone is most effective at altitudes above 4000 m at doses of 8−16 mg/d. Appropriately designed and powered trials are needed to obtain more evidence-based results on the dosage and timing of dexamethasone administration, and to provide optimized recommendations for the application of this powerful pharmacological tool.

## Background

Acute altitude illnesses and the impairment of cognitive and exercise performance are common consequences following rapid ascent to high altitude. Acute altitude illnesses primarily include neurological conditions, acute mountain sickness (AMS) and high-altitude cerebral edema (HACE), as well as a pulmonary condition, high-altitude pulmonary edema (HAPE) [1]. Acute altitude illnesses primarily affect people who are acutely exposed to high altitudes above 2500 m without sufficient acclimatization [2, 3]. AMS, a syndrome comprising headache and other symptoms, such as anorexia, nausea, vomiting, dizziness, and fatigue, is usually self-limiting but may progress to life-threatening HACE [2, 3]. HAPE is a potentially fatal consequence of acute high-altitude exposure, characterized by dyspnoea, cough, and exercise intolerance [4, 5]. The incidence of AMS depends on individual susceptibility, the maximum sleeping altitude, and the rate of ascent [6]. When ascending on foot, the risk of AMS increased from about 7% at 2200 m to 38% at 3500 m, and further to 52% at 4559 m [6, 7]. In a large cohort study, the incidence of severe AMS above 4000 m was 24% [8]. In contrast, with rapid passive ascent, by means including car, helicopter, or airplane, to altitudes of about 4000 m, the incidence may be above 80% [9, 10]. AMS can be effectively prevented by acclimatization [11]. However, when high-altitude work, rescue, or military operations require rapid ascent, there is often insufficient time for acclimatization [12]. Taking effective pharmacological measures to prevent or alleviate acute altitude illnesses is crucial for maintaining operational fitness and preventing morbidity and mortality. Moreover, cognitive and physical impairment after rapid ascent may impose several limitations on military or rescue operations. Maximal oxygen uptake (VO_2__max_) decreases by nearly 7% for every 1000 m of elevation gain. In one study, VO_2max_ was 40% lower at 6022 m than at sea level [13]. Acute exposure to high altitude also affects cognition in a dose-dependent manner, particularly at altitudes above 3000 m when arterial partial pressure of oxygen (PaO_2_) falls below 60 mmHg; the effects on cognition are less well understood than those on exercise performance [14]. An ideal pharmacological agent should take effect rapidly, be effective for all acute altitude illnesses at all altitudes, prevent cognitive and physical impairment, and have minimal adverse effects.

## Characteristics and main findings of the included studies

The existing literature on the use of dexamethasone (DEX) for the prevention of acute altitude illnesses and its effects on cognitive and physical performance during acute exposure at simulated or terrestrial altitudes above 3000 m was reviewed. These effects were evaluated based on altitude, exposure type (simulated or terrestrial altitude), DEX dose, and administration time. The databases PubMed, Science Direct, Embase, and the Cochrane Database of Systematic Reviews from their inception to March 2024 were searched using the following terms: “DEX” and “altitude or hypoxia” and “prevention or prophylaxis or incidence” and “placebo or control”. All randomized placebo-controlled trials that evaluated DEX effects on AMS, HACE, or HAPE prevention in healthy humans were included. We only considered studies with exposure to hypobaric (simulated or terrestrial) altitude above 3000 m, reporting the time and dose of DEX administration (doses > 1 mg/d), and applying clear diagnostic criteria for AMS, HACE, or HAPE diagnosis. AMS diagnosis had to be based on the Environmental Symptom Questionnaire (ESQ/AMS-C) score [15] and/or Lake Louise symptom score (LLSS) [16, 17]. The diagnosis of HACE and HAPE had to be based on clear symptoms, namely, altered mental status, ataxia, progression of AMS symptoms, or MRI for HACE, and dyspnoea, exaggerated loss of exercise performance, cough, gurgling, or chest X-ray showing patchy alveolar infiltrates with normal cardiac contours for HAPE. We only included studies if AMS, HACE, or HAPE incidence and/or severity were assessed during the first 2 days of the final altitude exposure. Main study characteristics and outcomes are presented in Table 1 [18–32]. We report effect sizes (ES) for effect differences between medication and placebo as Cohen’s d for means and Cohen’s h for proportions. ES < 0.2 are considered negligible, ≥ 0.2 and < 0.5 small, ≥ 0.5 and < 0.8 moderate, and ≥ 0.8 large.

**Table 1:** Characteristics of the 13 studies that met the inclusion criteria: preventive effects of dexamethasone and/or acetazolamide on acute mountain sickness or high-altitude pulmonary edema

Studies	Subjects Design	Altitude exposure	Ascent time	DEX dose	Treatment effects (mean ± SD): DEX (and/or ACZ or Budesonide) vs. Placebo (ES)	References
Johnson et al. (1984)	8 men, (20–26) years, crossover	4570 m, 42 h, simulated	0 h	4 mg every 6 h, start 48 h before altitude exposure	ESQ/AMS-C (score): 0.26 ± 0.08 vs. 1.09 ± 0.18^*^ ES: 6.0	[18]
Rock et al. (1989)	25 men, (18–32) years, crossover DEX 4 mg: 8 1 mg: 9	4570 m, 45 h, simulated	0 h	4 mg every 12 h, 1 mg every 12 h, start with altitude exposure	ESQ/AMS-C (score): 4 mg: 1.08 ± 0.25 vs. 2.71 ± 0.36^*^ ES: 5.3 1 mg: 1.93 ± 0.41 vs. 2.02 ± 0.35^#^	[19]
Subudhi et al. (2011)	20 (16 men), crossover AMS sus-ceptible: 6 Resistant: 14	4875 m, 9 h simulated	0 h	4 mg every 8 h (ACZ 250 m or placebo every 8 h), start 24 h before altitude exposure	AMS, LLS (score) AMS susceptible DEX: 1.3 ± 0.8^*^ ES: 3.8 ACZ: 1.3 ± 1.2^*^ ES: 2.8 Placebo: 4.0 ± 0.6 AMS resistant DEX: 0.7 ± 0.7^#^ ACZ: 1.1 ± 0.9^#^ Placebo: 1.1 ± 0.7	[20]
Maggiorini et al. (2006)	19 (16 men), HAPE susceptible: (43 ± 8) years DEX: 10 Placebo: 9	4559 m, 48 h active ascent	24 h	8 mg every 12 h, start with ascent	AMS, LLSS ≥ 4 (incidence): 30% vs. 89%^*^ ES: 1.3 HAPE: 0% vs.78%^*^ ES: 2.2	[21]
Rock et al. (1987)	16 men, (19 – 26) years DEX: 8 Placebo: 8	4300 m, 48 h, passive ascent	5 h	4 mg every 6 h, start 48 h before altitude exposure	ESQ/AMS-C (incidence): 31% vs. 60%^#^	[22]
Ellsworth et al. (1987)	47 (43 men), (18 – 52) years DEX: 17 ACZ: 15 Placebo: 15	4392 m, 1 h, active ascent	34.5 h	4 mg every 8 h (or 250 mg ACZ or Placebo every 8 h), start 24 h before ascent	AMS (AMS-index) DEX: 25%^*^ ES: 1.1 ACZ: 53%^#^ Placebo: 77%	[23–25]
Hackett et al. (1988)	15 men, (28 ± 1) years DEX: 7 Placebo: 8	4400 m, 12 h, passive ascent	1 h	2 mg every 6 h, start 1 h before ascent	ESQ/AMS-C (score): 2.6 ± 0.6 vs. 4.6 ± 1.0^#^	[26]
Zell and Goodman (1988)	32 (20 men), (18–49) years DEX: 8 ACZ: 8 DEX + ACZ: 8 Placebo: 8	3650 m, 48 h, passive plus active ascent	48 h	4 mg every 6 h (and/or 250 mg ACZ or Placebo every 12 h), start with ascent	ESQ/AMS-C (incidence) DEX, 12 and 24 h: 34% and 10%^#^ ACZ, 12 and 24 h: 30% and 0%^#^ DEX + ACZ, 12 and 24 h: 12% and 0%^#^ Placebo, 12 and 24 h: 51% and 38%	[27]
Ellsworth et al. (1991)	18 (11 men), (34 ± 4) years, crossover DEX: 10 ACZ: 8	4392 m, 1 h, active ascent	32 h	4 mg every 8 h (or 250 mg ACZ or Placebo every 8 h), start 24 h before ascent	ESQ/AMS-C (score): 0.26 ± 0.16 vs. 1.11 ± 1.02^*^ ES: 1.2 ACZ: 0.8 ± 0.8^#^	[28]
Bernhard et al. (1994	23 (15 men), (22–62) years DEX: 11 Placebo: 12	5334 m, 20 h, passive ascent	72 h	4 mg every 12 h, start 6 h before ascent	ESQ/AMS-C (score): 0.3 ± 0.3 vs. 2.1 ± 0.5^*^ (Day 3), 1.1 ± 0.2 vs. 2.5 ± 0.4^*^ (Day 4) ES (after 8 h and 20 h): 4.4	[29]
Hussain et al. (2001)	24 men, (27.8 ± 1.0) years DEX: 6 ACZ: 6 DEX + ACZ: 6 Placebo: 6	4578 m, 72 h, passive (+ 3 h active) ascent	24 h	4 mg every 12 h (and/or 250 mg ACZ or Placebo every 12 h), start 24 h before ascent	ESQ/AMS-C (score) DEX, 24 and 72 h: 0.72 ± 0.24 and 0.63 ± 0.20^#^ ACZ, 24 and 72 h: 0.95 ± 0.13 and 0.75 ± 0.20^#^ DEX + ACZ, 24 and 72 h: 0.23 ± 0.09^*^ and 0.18 ± 0.08^*^ ES: 3.6 and 5.0 Placebo, 24 and 72 h: 0.85 ± 0.23 and 0.78 ± 0.15	[30]
Zheng et al. (2014)	124 men, (18 – 35) years DEX: 39 Budesonide: 42 Placebo: 43	4200 m, 24 h, passive ascent	96 h	4 mg every 12 h, (or 200 μg Budesonide every 12 h), start 24 h before altitude exposure	AMS, LLS (score) DEX: 1.92 ± 1.72^*^ ES: 0.7 Budesonide: 1.64 ± 1.65^*^ ES: 0.9 Placebo: 3.42 ± 2.30	[31]
Chanana et al. (2022)	27 (15 men), (24–28) years DEX: 13 (7 men) Placebo: 14 (8 men)	3500 m, 74 h, passive ascent	1.5 h	4 mg every 12 h, start 24 h before altitude exposure	AMS, LLSS ≥ 3 (incidence) Day 1: 23% vs 21%^#^ Day 2: 0% vs 14%^#^ Greater benefits in females	[32]

*indicates statistical significance (*P* < 0.05) reported by the study authors or recalculated from the provided data; ^#^non-significant; in one study 2 dexamethasone doses were evaluated [19]. *ACZ* acetazolamide, *AMS-C* acute mountain sickness-cerebral score, *AMS Index* arbitrary symptom score [23, 24] similar to the LLSS, *DEX* dexamethasone, *ES* effect size, *ESQ* environmental symptom questionnaire, *LLSS* Lake Louise symptom score, *SD* standard deviation

Of the 197 articles identified in the literature search, 13 met the inclusion criteria and were selected for full analyses (Table 1). Altitude exposures varied from 3500 to 5343 m. Daily DEX doses varied from 2 to 16 mg. Three studies were performed at simulated altitude (hypobaric chamber) [18–20] and the others were conducted at terrestrial altitude [21, 22, 25–32]. The studies were heterogeneous in terms of sample size, sex distribution (5 studies included only men, and 8 studies involved both men and women), the time and dosage of medication administration, the type and duration of ascent (passive, active, or combined), altitude exposure conditions, and the reporting of the severity and/or incidence of AMS. While subjects without reported susceptibility to acute altitude illnesses were included in 11 studies, 2 studies included individuals susceptible to AMS or HAPE [20, 21]. The following subsections present the effects of DEX on AMS prevention in individuals with known or unknown susceptibility, on the prevention of HACE or HAPE, and the prevention of cognitive and exercise performance.

### DEX effects on AMS prevention

Of 11 studies (with unknown AMS susceptibility) evaluating the preventive effect of DEX (> 2 mg/d) within the first 24 h at the final altitude > 4000 m, 6 studies revealed significant results, of which 5 demonstrated large ES and one moderate ES. Five studies failed to show a significant DEX effect.

Of studies showing significant DEX effects, two studies involving young men were conducted using a controlled study design (double-blind crossover) at a simulated altitude of 4570 m. One used a preventive DEX dose of 4 mg every 6 h [18] while the other used 4 mg every 12 h [19]. Both demonstrated significantly lower AMS-C scores when pretreated with DEX compared to placebo (0.26 ± 0.08 vs. 1.09 ± 0.18 and 1.08 ± 0.25 vs. 2.71 ± 0.36; for both *P* < 0.05 and large ES). A large placebo-controlled study (total *n* = 47) compared the effects of DEX (4 mg every 8 h, starting 24 h before ascent) with those of acetazolamide (250 mg every 8 h) after active ascent to 4392 m [25]. This study confirmed the prophylaxis effect of AMS with DEX compared to placebo (AMS incidence: 25% vs. 77%; *P* < 0.05; large ES), while the preventive effects of acetazolamide were less pronounced and not statistically significant (53% vs. 77%). In addition, the authors stated that the side effects of acetazolamide (at this relatively high dosage of 750 mg/d) limited its usefulness as AMS prophylaxis [25]. In a follow-up cross-over study (*n* = 18), these authors reported AMS-C score (mean ± SD) reductions from 1.11 ± 1.02 with placebo to 0.80 ± 0.80 (not statistically significant) with acetazolamide (3 × 250 mg/d) and to 0.26 ± 0.16 with DEX (3 × 4 mg/d) (*P* < 0.05; large ES) [28]. A “non-rapid-ascent” study confirmed AMS prevention by DEX (2 × 4 mg/d) [29]. Twenty-three subjects ascended in 3 d to 5334 m; on day 3 (7 − 8 h after arrival at high altitude) the AMS-C score (mean ± SD) was 0.3 ± 0.3 and 2.1 ± 0.5 (*P* < 0.05; large ES), and on day 4, 1.1 ± 0.2 and 2.5 ± 0.4 (*P* < 0.05; large ES) in the DEX vs. placebo group [29]. Zheng and colleagues [31] compared the effects of DEX and inhaled budesonide on AMS prophylaxis during a slow (4 d) ascent to 3900 m. The authors showed similar preventive efficacy of both drugs (24% and 31% AMS incidence for budesonide and DEX, corresponding to moderate and large ES (*P* < 0.05), respectively) compared to placebo (60% AMS incidence) and reported favorable effects of budesonide on forced vital capacity and arterial oxygen saturation [31].

Of the studies that did not find significant DEX effects, two used 4 mg every six hours. In the study of Rock et al. [22], the AMS incidence was reduced from 60% with placebo to 31% with DEX (not statistically significant), likely indicating that the study was underpowered (lack of sufficient sample size to detect a true effect, if one exists). Another study compared the AMS incidence among 4 subgroups, including prophylactic use of DEX (4 × 4 mg/d), acetazolamide (2 × 250 mg/d), DEX plus acetazolamide, and placebo (*n* = 8 for each subgroup) [27]. Although the AMS incidence was 38% in the DEX group vs. 50% in the placebo group, the difference was not statistically significant. In addition to the small sample size, the relatively low maximum altitude of 3650 m might be one of the reasons for the lack of statistical difference. In this study, DEX plus acetazolamide was the most effective, reducing the AMS incidence to 12% (not statistically significant). Acetazolamide may have added to the efficacy of DEX because of prolonged treatment during the 2-day ascent and the 12 h stay at the final altitude of 3650 m before the first AMS recordings [27]. A similar study involving passive and active ascents to an altitude of 4578 m found that DEX (4 mg every 12 h) had no significant effect on preventing AMS [30]. However, DEX plus acetazolamide (250 mg every 12 h) was effective, likely related to the earlier start time of the pre-treatment (24 h before ascent), compared to the previous study, in which treatment began at the start of the ascent [27].

A study with rapid ascent to 4400 m by helicopter found that DEX at a dose of 2 mg every 6 h starting 1 h before ascent did not prevent AMS in male soldiers who engaged in heavy physical labor such as building snow walls and ice houses, and carrying heavy loads after arrival at altitude [26]. Because of a high AMS severity, the prevention trial was terminated after 12 h. Subsequent treatment with 4 mg DEX every 6 h effectively improved AMS, but symptoms increased again when treatment was discontinued [26].

In another study, participants were transported to 3500 m by airplane, and the AMS incidence was low on the first day in both the DEX (23%) and control group (21%). On the second day at altitude, AMS had completely disappeared in the DEX group but was still present (14%) in the control group (only in women) [32]. There were no statistically significant differences between DEX and placebo.

A single study examined the impact of DEX on the development of AMS in individuals who are susceptible to it and those who are not. A significant reduction in the LLS score was demonstrated in those susceptible to AMS (placebo vs. DEX: 4.0 ± 0.6 vs. 1.3 ± 0.8; *P* < 0.05; large ES), but not in those resistant to it [20].

In HAPE-susceptible (HAPE-S) individuals (subjects who had a history of previous HAPE), Maggiorini et al. [21] demonstrated significantly reduced AMS incidence (30% vs 89%; *P* < 0.05; large ES) with DEX (2 × 8 mg/d) pre-treatment before ascending to 4559 m within 48 h.

The average sample size of studies favoring DEX over placebo was *n* = 18 for the DEX and *n* = 19 for the placebo group, respectively. Values for studies without significant effect differences were *n* = 8 (DEX) and *n* = 9 (placebo).

### DEX effects on HACE prevention

No controlled studies evaluated the preventive effects of DEX on HACE development. However, AMS and HACE share some common pathophysiological mechanisms, suggesting that DEX may also be useful in HACE prevention.

### DEX effects on HAPE prevention

Maggiorini et al. [21] evaluated DEX pre-treatment for the prevention of HAPE (in HAPE-S subjects). None of the 10 subjects in the DEX group developed HAPE, whereas 7 of 9 subjects receiving placebo did (*P* < 0.05; large ES) [21].

### DEX effects on cognitive and exercise performance

Only one study performed maximal exercise testing and echocardiography at low and acute high altitude (4556 m) in HAPE-S subjects receiving DEX [33]. Besides protecting from AMS and HAPE, DEX slightly (but significantly) attenuated the relative (related to the individual body mass) decrease in VO_2__max_, which was 45% with DEX and 52% with placebo.VO_2max_ was evaluated in another subgroup of this study [21, 33] on the second day at high altitude [34]. While VO_2__max_ only fell to 70% of baseline with DEX, it decreased to 61% in the placebo group (*P* < 0.05) [34].

## Pharmacological properties and mechanisms potentially involved in DEX effects on the prevention of acute altitude illnesses

The potentially counteracting mechanisms of dexamethasone in the development of high-altitude illnesses are still poorly understood. A closer look at those mechanisms may provide a better foundation for future evaluation of the preventive potential for various types of acute altitude illnesses, including AMS, HACE, and HAPE, and the impairment of exercise performance and cognition. Hypoxia and some resulting pathological mechanisms are the primary causes for the development of acute altitude illnesses. They can be partially counteracted by DEX pre-treatment, and possibly more effectively than by acetazolamide. The following section aims to furnish an updated basis of the (patho) physiological mechanisms of DEX that are relevant for acute altitude illnesses.

### Pharmacological properties of DEX

Oral DEX is well absorbed in both healthy individuals and those suffering from acute infections [35, 36]. Its bioavailability (the rate and extent to which the drug or its metabolite enters the systemic circulation and accesses the site of action) amounts to about 80% and peak plasma levels are reached between 1 and 2 h after ingestion, although with a large interindividual variation. Its terminal half-life is about 7 h [35]. In the case of cerebral edema, the recommended doses are 6 to 16 mg/d, divided into 3 to 4 individual doses [36]. Based on the results of the presented studies, it seems reasonable to administer a dose of 2–8 mg of DEX every 6–12 h from the start of the ascent to help prevent high-altitude illness in emergencies.

### Potential DEX mechanisms for the prevention of AMS, HACE, and HAPE

**Fig. 1:**
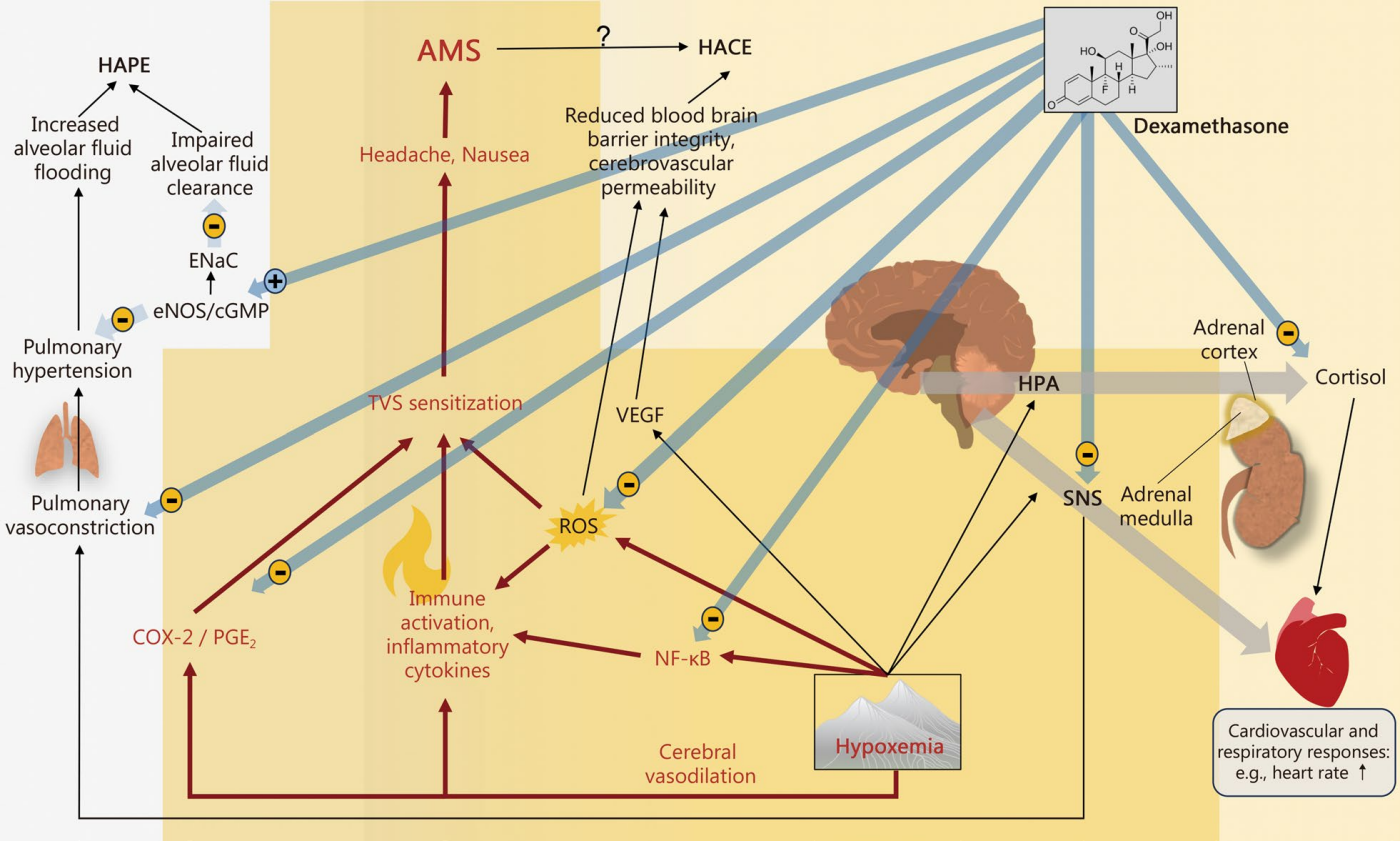
Suggested pathophysiology of AMS (HACE and HAPE) development and potentially counteracting mechanisms by dexamethasone. AMS acute mountain sickness, cGMP cyclic guanosine monophosphate, COX-2 cyclooxygenase-2, ENaC epithelial sodium channel, eNOS endothelial nitric oxide synthase, HACE high-altitude cerebral edema, HAPE high-altitude pulmonary edema, HPA Hypothalamic pituitary adrenal axis, NF-κB transcription factor nuclear factor-κB, PGE2 prostaglandin E2, ROS reactive oxygen species, SNS sympathetic nervous system, TVS trigeminal vascular system, VEGF vascular endothelial growth factor

Some meaningful mechanisms that are thought to mediate protection from acute altitude illness (with a focus on AMS) by DEX are depicted in Fig. 1.

DEX counteracts hypoxia-induced mechanisms that may cause AMS. It can likely also prevent HACE and HAPE, although optimized doses and treatment protocols remain to be identified. The development of AMS may be a consequence of hypoxia-induced cerebral vasodilation and vascular stretch, an increase in intracranial pressure, the induction of oxidative stress (as a result of excessive reactive oxygen species (ROS) production or insufficient ROS clearance), and inflammatory processes. These are associated with the upregulation of transcription factors and inflammatory regulatory transcription factor nuclear factor-κB (NF-κB), accompanied by the activation of cyclooxygenase-2 (COX-2) and the release of prostaglandin E2 (PGE2), ultimately leading to the sensitization of the trigeminal vascular system (TVS). Together, these effects are thought to provoke headache and other AMS symptoms [1]. DEX may exert protective effects by inhibiting COX-2 [37], NF-κB [38], ROS production [39], and overall promote anti-inflammatory effects by influencing multiple other signal transduction pathways [40]. Acute high-altitude exposure seems to be more importantly associated with the stimulation of the adrenal medulla than the sympathetic nervous system (SNS) [41]. DEX can inhibit the adrenal medullary response and its consequences, such as the increase in heart rate commonly observed during acute exposure to high altitude [41]. Notably, the well-established antiemetic effect of DEX [42] and its stimulating properties [43] may importantly counteract AMS. Finally, elevated blood glucose levels by DEX could increase the respiratory quotient and associated alveolar PO_2_, alleviating AMS.

HACE occurs as a consequence of increased cerebral vascular permeability and disruption of the blood-brain barrier, both of which can be triggered by ROS, inflammatory processes, and hypoxia-inducible factor (HIF)-related increases in vascular endothelial growth factor (VEGF) [1, 44]. DEX may prevent cerebral edema formation by inducing cerebral vasoconstriction, reducing cerebral blood flow, decreasing the production of cerebrospinal fluid, protecting tight junctions of the blood-brain barrier, and/or increasing pinocytotic activity in cells of the capillary endothelium [19, 45, 46].

HAPE is associated with uneven pulmonary vasoconstriction, overperfusion, increased capillary pressure, and stress failure in areas of little vasoconstriction [5]. The dysfunction of sodium transport leads to a decreased alveolar fluid clearance rate, which may contribute to the pathogenesis of HAPE [47]. DEX may prevent HAPE by stimulating cyclic guanosine monophosphate (cGMP) production in hypoxia and the related epithelial sodium channel (ENaC) activation, increasing the activity of endothelial nitric oxide synthase (eNOS), and possibly also by regulating the activity of SNS, at least in individuals prone to HAPE-S [21].

### Potential DEX mechanisms for maintaining cognitive and physical performance

The analgesic and anti-inflammatory properties of DEX are well established, but DEX (4.5 or 13.5 mg) did not improve perceived exertion or fitness or objectively assessed parameters of exercise performance in sea-level conditions [48]. Except for HAPE-S-individuals [33, 34], only very little information is available about DEX effects on cognitive and physical performance when acutely exposed to high altitude. Notably, the interaction between AMS and cognitive and physical performance is difficult to distinguish. In HAPE-S subjects, the observed minor improvements in aerobic capacity may have resulted from a sustained effect of DEX on maximal cardiac output and pulmonary oxygen diffusion, both of which improve oxygen delivery to exercising muscles [33, 34]. There are currently no controlled studies on the effect of DEX pretreatment on the maintenance of exercise performance during acute exposure to high-altitude environments. However, the prevention of acute altitude illnesses may indirectly benefit exercise performance. An early study reported positive influences of DEX (4 mg every 6 h) on cognitive performance and mood states in subjects exposed to 4300 m [49]. Another study found attenuated cognitive deficits compared to controls (better maintenance of reaction time for shape recognition) in individuals pretreated with DEX (2 × 4 mg) after acclimatization to 3700 m and subsequent ascent to 4800 m (to avoid severe AMS development) [50]. Thus, DEX may slightly improve cognitive parameters, but this could also be an indirect effect because of the prevention of acute altitude illnesses.

### Potential adverse effects of DEX

None of the studies included in the present analysis reported serious adverse effects. However, a literature review indicated a very low risk of harmful effects of corticosteroids during a short-term course (< 1 week) [51], which is the most relevant period for the prevention of acute altitude illnesses. Adverse effects of DEX include mental disturbances, water retention, and glucose intolerance [21, 46, 52]. Psychiatric adverse effects are common during systemic corticosteroid administration, causing severe adverse effects in 6% of patients and mild to moderate adverse effects in about 28% of patients [53]. While disturbances of cognition, mood, sleep, behavior, or even psychosis may all occur, euphoria and hypomania are the most common symptoms during short-term administration [53]. Subtle changes in mental state, such as a euphoric mood (as anecdotally reported by high-altitude climbers using DEX), may have gone undetected in the studies presented and could have masked altitude-related depression [54]. Unfortunately, we are not aware of any studies specifically evaluating the psychiatric adverse effects of DEX use for the prophylaxis of acute altitude illnesses. Such studies are urgently needed to ensure the safe use of DEX, particularly in people who are considered for operations at high altitude.

## Modulators of DEX effects

DEX effects vary depending on different conditions such as the severity of hypoxia exposure, the method and rate of ascent to high altitude, and the dose of DEX used for AMS prevention.

### Terrestrial versus simulated hypobaric hypoxia and by elevation

Variations in the methods of diagnosing AMS make a general assessment of the value of DEX for the prevention of AMS difficult. Considering those studies that used the same diagnostic criteria (ESQ/AMS-C), DEX pre-treatment was not effective at the 2 lowest terrestrial altitudes (3500 m and 3650 m) [27, 32] but was effective in all studies (*n* = 3) simulating more severe hypobaric hypoxia conditions [18–20] and using DEX doses above 2 mg/d. These findings may indicate a higher efficacy of DEX prophylaxis when ascending to higher simulated altitudes. Exercise and additional environmental stresses, in addition to hypoxia, are usually less pronounced in simulated altitude studies, and the development of AMS, HACE, or HAPE is less likely [55–57]. A possible explanation for the seemingly increasing efficacy of DEX prophylaxis with increasing altitude may be the higher probability of acute altitude illnesses (concerning AMS [9] and especially HACE and HAPE, making the prophylactic efficacy of DEX more apparent [2, 58]. This is of practical and clinical relevance because not only does the incidence but also the severity of acute altitude illnesses increase at higher elevations.

### Method and rate of ascent

Rapid ascent and poor acclimatization to high altitude are major risk factors for the development of acute altitude illnesses [59]. Active ascent involving heavy exertion is more likely to cause more severe acute altitude illnesses than passive ascent to a comparable altitude [55]. This may also help to explain why the preventive efficacy of DEX use was statistically significant [18–20, 29, 31] or of clinical relevance [22, 26] in all studies, except one (at the relatively low altitude of 3500 m) [32], with a passive ascent to high altitude. The average ascent time in the studies included in this review was 26 h (range: 0–96 h) (Table 1). The importance of the ascent time remains unclear, since the use of DEX prevented the development of AMS in 3 of 6 studies with ascent times less than 24 h [18–20] and in 4 of 7 studies with ascent times longer than 24 h [23, 28, 29, 31], including both passive and active ascents.

The study with ascent by helicopter [26] is the only one reflecting true conditions of rapid ascent, followed by immediate and exhausting work at high altitude. These conditions are highly relevant for rescue operations. The DEX dose, 2 mg during the initial 4 h working at high altitude, followed by 2 mg every 6 h, was probably too low to prevent AMS under these stressful conditions. Although differences between DEX and placebo effects were not statistically significant (AMS-C scores: 2.6 vs. 4.6; *P* = 0.09), a large ES (2.4) strongly indicates that this study was underpowered.

Although there appears to be a tendency for greater benefits of DEX prophylaxis when passively ascending to high altitude (in particular when no strenuous work is carried out at high altitudes), the limited data do not allow for conclusions regarding the impacts of rate or method of ascent on the efficacy of DEX to prevent AMS.

### Effects of DEX dose

In studies reporting AMS-C scores [18, 19, 26, 28–30], the effectiveness of DEX in preventing AMS generally increased with increasing dose.

Of 4 studies using the highest DEX doses (16 mg/d), 2 found significant preventive effects regarding the development of acute altitude illnesses, but one of these included HAPE-S subjects. One of the 2 studies that reported DEX to be ineffective was conducted at a relatively low altitude (3650 m) [27], and the other one (*n* = 8 DEX + 8 placebo) [22] may have been underpowered. The three studies that used DEX 12 mg/d and 3 of the 6 studies using 8 mg/d found prophylactic efficacy. One of the studies that reported DEX administration 8 mg/d to be ineffective was performed at a relatively low altitude (3500 m) [32], and the other 2 were possibly underpowered [26, 30]. No preventive efficacy was observed in the only study using DEX 2 mg/d [19]. These findings suggest a dose–response relationship, indicating that daily doses above 8 mg DEX are more effective for the prevention of acute altitude illnesses than lower doses.

### Timing, divided doses, and co-administration with acetazolamide

Start time had no discernible effect on efficacy, nor did dividing doses. Because of the long half-life of DEX, the drug begins to accumulate with the first dose and will continue to do so until a steady-state equilibrium is reached. Thus, larger initial (and subsequent) doses will more rapidly result in higher drug concentrations, which may be desirable for rapid ascents to high altitude. However, the included studies do not allow for drawing conclusions regarding this aspect as all studies except the one of Hackett et al. [26] (starting dose of 2 mg) and the one of Maggiorini et al. [21] (starting dose of 8 mg) used an initial DEX dose of 4 mg. The combination of DEX plus acetazolamide was similarly or more effective than DEX alone and may therefore be considered for AMS prophylaxis when rapidly ascending to high altitude.

Because the methods of the studies were extremely heterogeneous, a reliable assessment of the efficacy of DEX is not possible. The majority of studies had low statistical power. AMS has been assessed using different diagnostic tools, such as AMS-C and LLS scores. DEX doses were not adjusted to the individual body mass in any of the studies. This may explain some of the variability in the efficacy of DEX among the studies. Most studies were conducted with adult male subjects, which limits their applicability to women and older individuals. Lastly, there are very limited data regarding the effects of DEX on cognitive and exercise performance at high altitude.

## Conclusions

The presented data indicate a better efficacy of DEX at higher altitudes (i.e., > 4000 m) at daily doses from 8 to 16 mg when rapid ascent is necessary. We recommend future evaluation of DEX dosing related to the individual body mass and to determine inter-individual differences and relevant vulnerabilities to maximize prevention of acute altitude illness and alleviate hypoxia-induced impairment of cognitive and exercise performance. In particular, potential adverse effects of DEX, the interplay of medication with psychological stress (and the activation of stress pathways in parallel to altitude exposure), and stress-related psychiatric and other diseases require attention. The prophylactic use of DEX is only intended to support rapid ascents to high altitude in emergency cases, and we do not recommend its use in trekking and high-altitude climbing, where appropriate acclimatization remains the measure of choice to prevent acute altitude illnesses.

## Data Availability

Not applicable.

## Abbreviations

ACE: Angiotensin-converting enzyme
ACZ: Acetazolamide
AMS: Acute mountain sickness
cGMP: Cyclic guanosine monophosphate
COX-2: Cyclooxygenase-2
DEX: Dexamethasone
ENaC: Epithelial sodium channel
eNOS: Endothelial nitric oxide synthase
ES: Effect size
ESQ/AMS-C: Environmental Symptom Questionnaire/acute mountain sickness-cerebral score
HACE: High-altitude cerebral edema
HAPE: High-altitude pulmonary edema
HIF: Hypoxia inducible factor
HPA: Hypothalamic pituitary adrenal axis
LLSS: Lake Louise symptom score
NF-κB: Nuclear factor 'kappa-light-chain-enhancer' of activated B-cells
PaO_2_: Partial pressure of arterial oxygen
PGE2: Prostaglandin E2
PO_2_: Partial pressure of oxygen
ROS: Reactive oxygen species
SD: Standard deviation
SNS: Sympathetic nervous system
TVS: Trigeminal vascular system
VCO_2_: Carbon dioxide production
VE: Minute ventilation
VEGF: Vascular endothelial growth factor
VO_2__max_: Maximal oxygen uptake
